# Short-Course Treatment With Imipramine Entrapped in Squalene Liposomes Results in Sterile Cure of Experimental Visceral Leishmaniasis Induced by Antimony Resistant *Leishmania donovani* With Increased Efficacy

**DOI:** 10.3389/fcimb.2020.595415

**Published:** 2020-11-10

**Authors:** Sandip Mukherjee, Supratim Pradhan, Souradeepa Ghosh, Shyam Sundar, Shantanabha Das, Budhaditya Mukherjee, Syamal Roy

**Affiliations:** ^1^ Infectious Disease and Immunology Division, CSIR-Indian Institute of Chemical Biology, Kolkata, India; ^2^ School of Medical Science and Technology, Indian Institute of Technology, Kharagpur, India; ^3^ Department of Medicine, Institute of Medical Sciences, Banaras Hindu University, Varanasi, India; ^4^ National Institute of Pharmaceutical Education & Research, Kolkata, India

**Keywords:** visceral leishmaniasis, antimony resistance, imipramine, liposome, efficacy

## Abstract

Previously we have shown that long term oral treatment of tricyclic-antidepressant-drug, imipramine, against experimental visceral leishmaniasis, results in clearance of organ parasites, regardless of input infection, either with antimony-sensitive (Sb^S^) or antimony-resistant (Sb^R^) *Leishmania donovani* (LD) clinical isolates. Although continuous imipramine monotherapy for 28 days (5 mg/kg) results in significant clearance of organ parasites in both Sb^R^ and Sb^S^LD infected hamsters, the dose for the sterile parasite clearance from visceral organ is comparatively higher (10 mg/kg) and shows signs of toxicity. Hence, to reduce the toxicity, we encapsulated imipramine in squalene-phosphatidylcholine (SP) liposome (Lip-Imi) and tested its efficacy for a short-course treatment (10 days) in the animal model of visceral leishmaniasis. We observed a significant reduction of hepatic toxicity coupled with sterile parasite clearance in case of this short-course treatment of Lip-Imi, which is absent with free Imi treatment. This also correlates with significant increase in serum availability of imipramine in case of Lip-Imi treatment due to sustained release. Clearance of parasite was coupled with the polarization of antileishmanial immune repertoire from Th2 to Th1 after treatment with Lip-Imi in both Sb^R^LD and Sb^S^LD infected mouse models of LD infection. This study showed that imipramine is effective against both Sb^S^LD and Sb^R^LD at a significantly lower dose with reduced time course of treatment without any toxic side effects, when encapsulated in SP-liposome. Thus, the drug has the potential to be repurposed for the treatment of Kala-azar.

## Introduction

Leishmaniasis caused by different species of protozoa of genus *Leishmania* that are transmitted by Phlebotomine sandfly vector. LD causes visceral leishmaniasis (VL) in the Indian subcontinent, Asia, and Africa and is usually fatal if left untreated  (Lukes et al., 2007; Clem, 2010). The problem of drug resistance is very common in treating infectious diseases and leishmaniasis is not an exception (Sundar, 2001). Long treatment regime, too long half-life, large drug dose, rampant uses of drugs are the key factors that regulate resistance induction (Croft and Coombs, 2003). Absence of effective vaccines makes chemotherapy the only treatment option. Pentavalent antimonials (Sb^V^) like sodium stibogluconate (SSG) and meglumine antimoniate remain the first line therapy for VL for over six decades and are still in use in many parts of the world, where resistance to Sb^V^ is not prevalent (Haldar et al., 2011). Although, few alternative treatments like Amphotericin B (AmB), miltefosine, paromomycin, and lipid-conjugated-formulations of AmB are available, they also suffer from multiple limitations (Olliaro et al., 2005; Croft et al., 2006b; Sundar and Chatterjee, 2006; Maltezou, 2010). Approaches to overcome antimonial resistance include use of alternative drugs (Croft et al., 2006a), short-treatment regime (Sundar et al., 2000a) and better drug delivery system (Black et al., 1977; Valladares et al., 2001) to avoid side effects and resistance induction. Sometimes drugs show very less effectiveness in its tolerance level and the effective dose becomes toxic to host. Thus, efforts are made to minimize the above-mentioned factors which would lead to a better drug for effective chemotherapy. Previously, we reported that antidepressant imipramine is a potent antileishmanial agent in hamster model (Mukherjee et al., 2012)and it has been also shown to be effective against other *Leishmania* species (Andrade-Neto et al., 2016), thus qualifying as a potential candidate it can be used across the phylum. Interestingly, a structural study has reported some imipramine-analogues might also be effective against LD (Pandey et al., 2016). However, all the recommended dose-range of imipramine associated with treating depression (100–200 mg daily) for enuresis (10–75 mg daily) (Goodman, 1990), and for the children (daily doses of 25–50 mg to maximum 100 mg), are associated with some events of toxic effects (Saraf and Klein, 1971). However, very recently its lower dose has been approved by FDA and is already available commercially without any such event (Fayez and Gupta, 2020). We have previously reported that oral administration of imipramine, 5 mg/kg/day, for 10 days results in significant organ parasite clearance in infected hamsters. However, to obtain sterile cure of organ parasite burden we need to apply a higher dose of 10 mg/kg/day for a time period of 4 weeks which is a long treatment regime (Mukherjee et al., 2012). Thus, to reduce duration, we trap imipramine in squalene-phosphatidylcholine-liposome (Lip-Imi). Squalene a linear triterpene, has a very large spectrum in clinical use (Kamimura et al., 1992; Kohno et al., 1995). It is also used in the adjuvant formulations in conjunction with some surfactants and administered along with vaccine to stimulate the immune system (Klucker et al., 2012). Squalene emulsion with sustained release property, shows a prolonged effect at specific site (Fox, 2009). Phosphatidylcholine is a very important lipid component of liposomal drug delivery system. The liposomal formulation of AmpB, i.e., ambisome is in human use (Hay, 1994). Liposomal formulation of antimonials are also more effective in leishmaniasis (Alving et al., 1978; Borborema et al., 2016). Recently, antidepressant-Sertraline delivered in phosphatidylserine liposomes has shown effectiveness in an experimental model of VL (Romanelli et al., 2019).

We tested the efficacy of imipramine entrapped liposome formulation in *in vitro* and *in vivo* mouse and hamster model of leishmaniasis. Here we showed that liposomal formulation of Lip-Imis more potent antileishmanial candidate than imipramine alone. We observed a drastic decrease of imipramine dose in this new formulation to clear ∼100% organ parasite load in significant shorter treatment regime compared to the previously reported oral delivery, with increased T-cell proliferation, associated with Th1 polarization, and no sign of toxicity as observed previously with long regime of imipramine monotherapy.

## Materials and Methods

### Animals

BALB/c mice (*Mus musculus*) and hamsters (*Mesocricetus auratus*) were maintained and bred under pathogen free conditions.Animal use was approved by the Institutional Animal Ethics Committees of Indian Institute of Chemical Biology, Kolkata, India. All experiments were performed according to the National Regulatory Guidelines issued by CPSEA (Committee for the Purpose of Supervision of Experiments on Animals, IICB/AEC-15-2008, 10.06.2008), Ministry of Environment and Forest, Government of India.

### Parasite

Two different well characterized clinical LD isolates were used in this investigation. The details of the patients and their treatment profiles from whom LD parasites were derived have been published previously  (Mukhopadhyay et al., 2011). Clonal population of LD parasite MHOM/IN/83/AG83 (AG83) is antimony sensitive (Sb^S^) and strain MHOM/IN/09/BHU575/0 (BHU 575) is antimony resistant (Sb^R^). LD promastigotes were cultured in M199 medium (Sigma Aldrich, St. Louis, MO) supplemented with 10% heat inactivated FBS (Gibco), 100 IU/mL of penicillin, and 100 µg/mL of streptomycin (Gibco) in a 22°C room  (Mukhopadhyay et al., 2011).

### Preparation of Liposomes and Drug Solution for Drug Assays

Liposomes were formulated by addition of chloroform and methanol (9: 1 v/v) to a mixture of phosphatidylcholine (Avanti Polar lipids), squalene (Sigma) either with or without imipramine. The lipid solution was dried in dry nitrogen stream with continuous rotation. The dried mixture was then kept in vacuum desiccator for some time and then hydrated using rehydration buffer. Vesicles were prepared by sonicating the hydrated mixture of 1 ml of the above buffer for 10 min in an ice bath using a Misonix sonicator with a needle probe. The molar ratio of squalene to phospholipid was 1:1.5. Imipramine hydrochloride (Sigma Aldrich, St. Louis, MO) solutions were prepared at 1 mg/ml in PBS (Sigma Aldrich, St. Louis, MO), followed by sterile filtration using 0.22 µM filters (Millipore) as and when required.

### Size Distribution of Liposomes

The mean diameter and particle size distribution of liposomes were determined by an optical particle size analyzer by intensity using differential light scattering (DLS) method (López et al., 1999). Incorporation (% entrapment) of the imipramine in the liposome was determined with the optical density (OD) measurement. Imipramine shows a sharp peak at 250 nm. Analysis was performed on supernatants following PBS washes. The percentage of entrapment was calculated as follows:

% entrapment = [(total amount of imipramine added -amount of imipramine recovered in supernatant)/ total amount of imipramine added]×100

(Badiee et al., 2007)

### Cryo-EM

Cryo-EM was done as per protocol (Rasch et al., 2012). Briefly, specimen was developed on C-flat holey carbon film TEM grids with 1.2 μm diameter holes and 1.3 μm spacing. Squalene vesicles are imaged in the holey stretch of the carbon film.

### Infection, Administration of Liposomal Imipramine, and Determination of Parasite Burden in Mice and Hamster

Six weeks old BALB/c mice or hamsters were infected with either BHU 575 (Sb^R^LD) or Ag83 (Sb^S^LD) (5 × 10^6^ parasites in 100 μL) via intracardiac routes (Mukherjee et al., 2012). After 6 weeks of infection, animals were divided in six groups. Group-I received saline, Group-II received empty liposome, Group-III-IV received different doses of Lip-Imi (10 and 20 mg/kg) i.v.at days 1, 4, 7, and 10 and group-V received imipramine (10 mg/kg/day) orally. In case of mice liposome was injected through tail vein and for hamsters, liposomes injected through cephalic veins (Fiat et al., 1990). Two days after the completion of treatment, mice were sacrificed to determine splenic and hepatic parasite burdens by stamp smear method (Mukherjee et al., 2012).

### Determination of Serum Imipramine Level

Blood was collected from hamsters and mice (Basu et al., 2005) and kept overnight at 4°C; serum was prepared by centrifugation. High-performance liquid chromatography (HPLC) methods (Rezazadeh and Emami, 2016) were employed to extract imipramine from serum. Extracted imipramine was quantified by spectroscopic technique as imipramine shows a sharp peak at 250nm.

### T-Cell Proliferation Assay

Splenocytes from different experimental groups of mice were prepared and then suspended in complete RPMI medium. Assay was performed in presence or absence of SLA (5 µg/ml). ConA (Sigma Aldrich, St. Louis, MO) was used to induceproliferation, the mitogen was added at a concentration of 5 µg/mL (Basu et al., 2005). Cells were treated with MTT (0.5 mg/mL) 4 hrs. before harvest and incubated again at the same condition for 4 more hrs. (Mukherjee et al., 2012). MTT crystals were then solubilized using Isopropanol-HCl mixture (0.04%) and the absorbance at 570 nm was read at an ELISA plate reader.

### Cytokine Analysis by ELISA

Various cytokine levels in the murine splenocytes culture supernatant were measured using a sandwich ELISA Kit (BD Biosciences, USA) as per manufacturer’s protocol.

### Liver and Kidney Function Assay

Serum levels of alanine transaminase (ALT), aspartate transaminase (AST), alkaline phosphatase (AP) and creatinine were determined in LD infected hamster model using the kit of Span Diagnostics Ltd. (India) and as per manufacturer’s instruction.

### Survival Kinetics

Survival kinetics was studied in hamster model as BALB/c have been reported to able to mount an antileishmanial cellular immune response which can control further parasite replication whereas infection in hamster closely resembles human disease (Melby et al., 2001). To evaluate long-term therapeutic ability, normal hamsters, infected hamsters and Lip-Imi (10 mg/kg) and oral Imi (10 mg/kg) treated (for 10 days) infected hamsters (10 hamsters per group) were used to study survival kinetics (Mukherjee et al., 2012). In short, 6-week-old hamsters were infected, and the infection was allowed to proceed for another 6 weeks, i.e., before initiating any treatment. Treatment (10 mg/kg Lip-Imi and 10 mg/kg Imi) was given as mentioned earlier.

### Histological Studies

Liver from the hamster were collected and fixed in 10% formalin (Merck) and embedded in the paraffin. To study the microarchitecture, tissue sections were stained with H&E. Detailed structural analytic photomicrographs were taken with a Nikon Eclipse E200 microscope.

### Statistical Analysis

The *in vitro* cultures were set in triplicates and the animal experiments were carried out with 5–6 mice per group unless or otherwise mentioned. Data shown are representative of at least three independent experiments and are expressed as mean ± SD. Student’s *t*-test was employed to assess the statistical significances of differences among pairs of datasets with only *P* value <0.05 considered to be significant. Values were considered extremely significant (p < 0.001) (represented as ***), very significant (p = 0.001–0.01) (represented as **), or significant (p = 0.01–0.05) (represented as *) as indicated. Error bars indicate means ± SD. Data were analyzedusing Prism 6.0 (GraphPad Software, San Diego, CA).

## Results

### Quantification of Drugs Entrapment in Liposome

**Figure 1:**
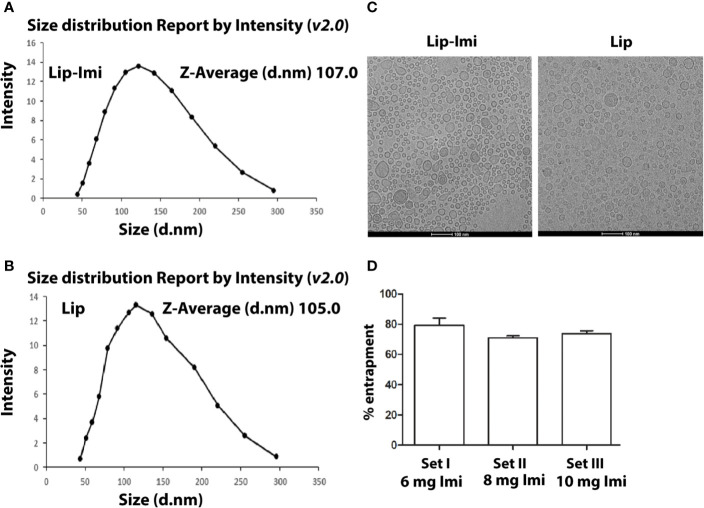
**(A**, **B)** Measurement of size of Imipramine entrapped PC-Squalene liposomes and empty liposome by differential light scattering (DLS) method. Liposomes are prepared, diluted and scanned under optical particle analyzer. **(C)** Representative figure of Cryo-EM structure of Lip-Imi (panel1) and empty Imi (panel2). **(D)** Measurement of the amount of liposome entrapped imipramine by the quantification of supernatant imipramine. Set I, Set II and Set III represents % liposomal entrapment from 6, 8, and 10 mg imipramine, respectively. Each set represents independent quantification experiment.

The liposome formulations were heterogeneous in size and their diameter ranging from 40 to 300 nm. Imipramine shows a sharp peak at 250 nm. The mean diameters calculated by particle size analyzer were 107 and 105 nm (Mean ± SD, n = 3) for Lip-Imi and empty liposome (**Figures 1A, B**) respectively,with individual Cryo-EM structure represented in **Figure 1C**. Thus, entrapment of the imipramine within liposomes had no apparent effect on the size or on the multilamellar structure. The entrapment of imipramine in the Lip-Imi was 74 ± 4% (mean ± SD, n = 3) as represented in the **Figure 1D**. The concentration of the imipramine in the liposomes was adjusted to 1.25 mg/mL after purification and calculation of % entrapment.

### Liposomal Imipramine Resolves LD Infection in Mice

**Figure 2:**
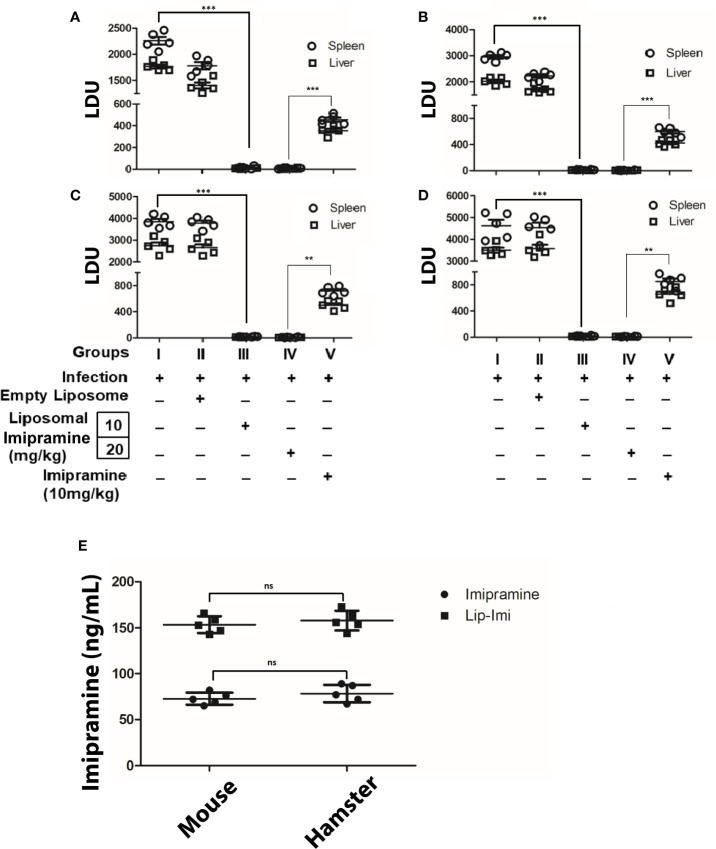
Six weeks old mice/hamsters were infected with either BHU 575 **(A**, **C)** or Ag83 **(B**, **D)** LD parasites and infection was allowed to establish for next 8 weeks. Eight-week infected mice/hamsters received the following treatment: Saline (Group I), vesicles (Group II), Lip-Imi (10 mg/kg) (Group III), Lip-Imi (20 mg/kg) (Group IV), respectively at days 1, 4, 7, and 10 and group V received 10 mg/kg/day of imipramine orally for 10 days. Two days after the last treatment, mice/hamsters were sacrificed, and the hepatic and the splenic parasite load was determined by stamps-smear method and the by the limiting dilution method. Total parasite load in each organ is expressed in LDU unit. 1 LDU = amastigote per nucleated cell × organ weight in milligram. Total concentration of imipramine in the serum of mice and hamster after the completion of treatment of imipramine and liposomal imipramine **(E)**. Results are presented as means ± SD, with statistical significance being determined with respect to infected control or between Lip-Imi and oral imipramine treatment. ***p < 0.001 and **p = 0.001–0.01 and NS-representing non-significant.

BALB/c mice (6 weeks old, ~25 g) and hamster (6 weeks old, ~40 g) were infected with either Ag83 (Sb^S^LD) (**Figures 2A, C**) or BHU575 (Sb^R^LD) (**Figures 2B, D**) metacyclic promastigotes. Liposomal-imipramine treatment was carried out after 6^th^ week of infection, with two different doses (10 and 20 mg/kg), and was administered intermittently (Basu et al., 2005) through tail vein at days 1, 4, 7, and 10. Two days after last drug treatment, antileishmanial potency was assessed in terms of hepatic andsplenic parasite burden. During the experiment, all the animals remained healthy with no marked effect on body weight. Imipramine doses of (10 and 20 mg/kg) trapped in liposome results in significant suppression of hepatic/splenic parasite burden in infected mice and hamsters, irrespective of the nature of input infection. However, no significant difference in terms of parasite clearance was observed between the doses. Absence of parasites in the spleen was further confirmed by culturing spleen specimens in transformation medium (M199) at 22°C for 96 h. Notably, 10 days of continuous oral treatment with free-imipramine (10 mg/kg) also cleared significant parasite burden as compare to infected controls, but parasite burden was significantly more compare to both the doses Lip-Imi treatment (**Figure 2**). Importantly, serum concentration of imipramine at the end of the experiment was found to be significantly higher in the liposomal imipramine treated animals (both hamster and mouse, with no significantinter species variation), suggesting sustained release of the drug might result in higher efficacy of parasite clearance as compare to free-imipramine treatment (**Figure 2E**).

### Liposomal Imipramine Treated in Infected Mice Induces Host Protective Cytokines From Splenocytes

**Figure 3:**
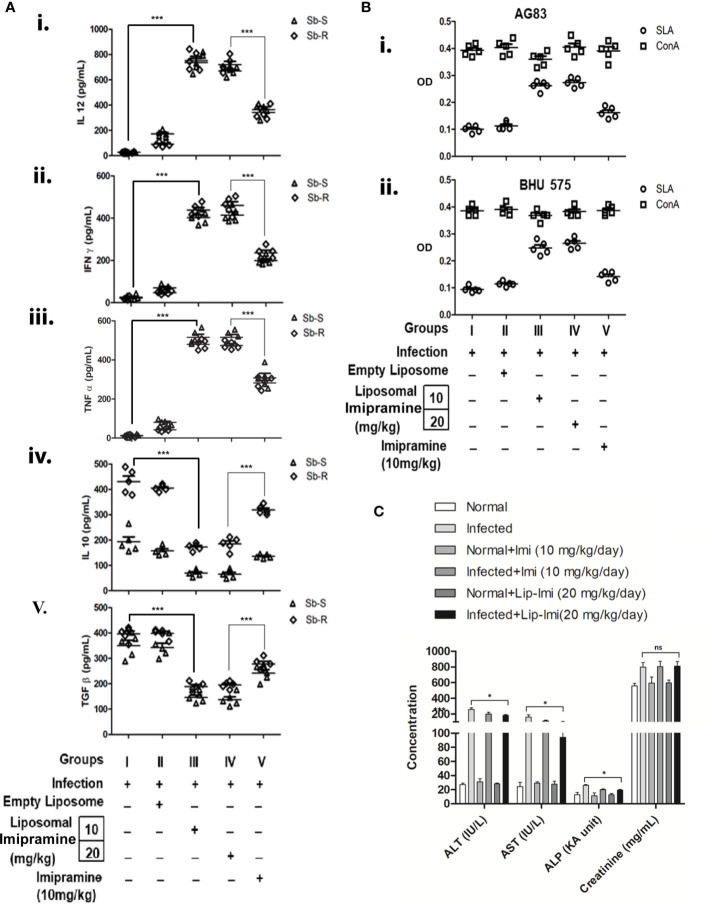
**(A)** *Leishmania donovani* (AG83 or BHU575)-infected and treated mice were sacrificed, and splenocytes were isolated and incubated with 5 µ/mL of SLA for 48 h. Levels of cytokines (i) IL-12, (ii) IFN-γ, (iii) TNF-α (iv) , and IL-10. (v) TGF-β in culture supernatants were determined by ELISA. Results are representative of one of three individual experiments. **(B)** Imipramine treatment favors expansion of antileishmanial T-cells. Splenocytes isolated from Sb^S^LD **(B**, I**)** and Sb^R^LD **(B**, II**)** LD infected (Group I), vesicles (Group II), Lip-Imi (10 mg/kg) (Group III), Lip-Imi (20 mg/kg) (Group IV), and orally 10 mg/kg for 10 day (Group V) drug treated mice were stimulated with SLA (5 µg/mL) (○) and non-specific mitogen ConA (5 µg/mL) (□), and the resulting proliferation of splenocytes was assayed using MTT cell viability assay. **(C)** The hepatic enzyme (ALT, alanine transaminase; AST, aspartate transaminase; ALP, alkaline phosphatase) and serum creatinine level in untreated uninfected, untreated infected, and imipramine treated infected hamsters.For A, results are presented as means ± SD, with statistical significance being determined with respect to infected control or between Lip-Imi and oral imipramine treatment. ***p < 0.001. For **(B)**, experiments were repeated thrice one representative data is shown. For C, results are presented as means ± SD, with statistical significance being determined with respect to normal uninfected or LD infected animals in presence or absence of Lip-Imi treatment. *p = 0.01–0.05.

To further compare the efficacy of effect of liposomal imipramine, we compare the effect of imipramine and Lip-Imi on host immune response. Host protective cytokine (IL-12, TNF-α, and IFN-γ) and disease promoting cytokine (IL-10 and TGF-β) productions from the splenocytes of infected and liposomal-imipramine treated infected mice was assayed. As expected splenocytes of infected (Group-I) and empty liposome treated infected mice (Group-II) responded poorly toward SLA stimulation and produced basal level of host protective cytokines, although significantly higher production was observed in case of liposome treated group. On the other hand, splenocytes of infected mice receiving liposomal imipramine at the dose 10 mg/kg (Group-III) and 20 mg/kg (Group-IV) in response to SLA produced significant amount of host protective cytokines, which plateaued at the highest concentration of liposomal imipramine used.Splenocytes of infected mice receiving oral imipramine (Group-V) also produced host protective cytokines which is significantly less to Group-III, IV mice (**Figures 3A** i, ii, and iii).

In terms of disease promoting cytokine profile, Group-I mice infected with Sb^R^LD resulted in more than two-fold higher IL-10 production as compare to their sensitive counterparts. Interestingly this higher level of IL-10 is also reflected in Sb^R^LD infected receiving only liposome (Group-II). Mice receiving liposomal imipramine produced significantly less IL-10 (**Figure 3A**, iv). Interestingly mice infected with Sb^R^LD induced higher IL-10 level as compared to those infected with Sb^S^LD as observed previously (Mukhopadhyay et al., 2011; Mukherjee et al., 2012). Like IL-10, this higher frequency was also observed in case of TGF-β production in Sb^R^LD infected mice and corroborates with a previous report (Guha et al., 2014) (Mukherjee et al., 2020). TGF-β production also decreased significantly upon liposomal-imipramine treatment (**Figure 3B**, v). There was no difference noted between Group-III &Group-IV. Oddly enough, TGF-β level remained significantly higher in orally imipramine treated group (Group-V) as compared to liposomal imipramine treated groups (Group III/Group IV) (**Figure 3A**, v).

### Imipramine Treatment Favors the Expression of Antileishmanial T-Cell Repertoire of Infected Mice

To study the status of antileishmanial T-cell repertoire in infected and imipramine treated infected mice, isolated splenocytes, fixed concentrations of SLA and ConA were used to stimulate splenocytes (Basu et al., 2005). Splenocytes of Group-I and Group II mice failed to mount any antileishmanial immune response but responded well to non-specific mitogen ConA regardless of the input parasites for infection (**Figures 3B**, I, II). The SLA specific proliferation was significantly improved in Group-III and Group-IV and showed almost similar proliferation. Group-V mice also showed moderate level of response. The antileishmanial T-cell response was essentially similar regardless of the phenotype of input infection. The response to the non-specific mitogen ConA remained unaltered in infected and in imipramine treated animals regardless of the imipramine-dose (**Figure 3B**).

### Hepatic Enzymes and Serum Creatinine

In terms of toxicity, treatment of naïve animals with Lip-Imi (10 and 20 mg/kg) does not resulted in any significant alteration of liver enzyme and creatinine level as compare to normal uninfected animals (**Figure 3C**), which clearly suggests that there is no additive adverse hepatic toxicity due to Lip-Imi treatment. However, level of hepatic enzymes and creatinine was significantly increased in serum in response to LD infection as compare uninfected control. Furthermore, treatment with Lip-Imi results in slight but significant suppression of hepatic enzyme level as compare to infected control group. However, serum creatinine level remained unaltered throughout the treatment course as compare to infected control group.

### Infected Hamsters Treated With Liposomal Imipramine Survives for Long Run

**Figure 4:**
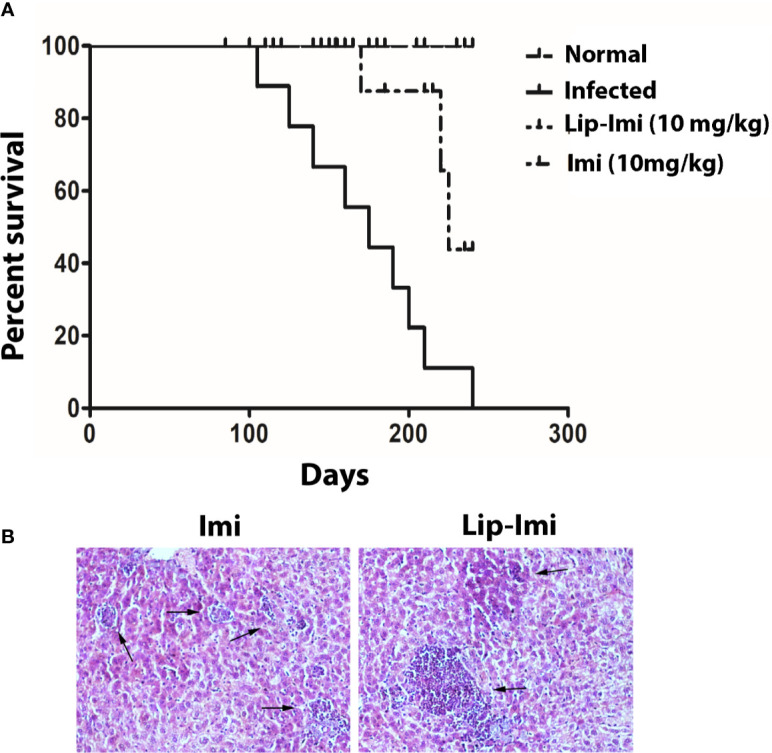
**(A)** Long term survival of normal, BHU 575 infected, oral 10 days imipramine treated and Lip-Imi (10 mg/kg) treated infected hamsters in terms of % survival. 20 hamsters from each group were used for the study. **(B)** Photographic representation (×40), of enhanced granuloma development (black arrow, right panel) in Lip-Imi (10 mg/kg) treated hamsters as opposed to immature granuloma (black arrow, left panel) formation in Imi (10 mg/kg) treated hamsters following infection with BHU575.

Survival and evolution of granuloma formation was compared in Lip-Imi and free-Imi treated infected hamsters. It was observed infected hamsters started dying around 107 days p.i. and all were dead by 240 days, i.e., termination of the experiment. As compare to oral Imi-treatment, Lip-Imi treated hamsters were completely protected and found to be survived throughout the experiments just like uninfected hamsters (**Figure 4A**). Hamsters treated orally with Imi for days, started showing mortality around 211 days. Increased survival rate of Lip-Imi treated hamsters also corroborates with high frequency of lymphocyte infiltration in periportal area and the presence of fair number of mature and uniform granuloma (**Figure 4B**, right panel), as oppose to only Imi-treated hamsters where presence of few parasitized immature granuloma could still be detected (**Figure 4B**, left panel).

## Discussion

Efficient short-course treatment of leishmaniasis is a basic research question now a day (Gradoni et al., 1995) due to its cost effectiveness and low impact in resistance development. The major obstacle of the existing treatment options for VL are the emergence of drug-unresponsive strains (Sundar and Murray, 1996; Sundar et al., 2000b) and cost of the treatment (Faraut-Gambarelli et al., 1997; Thakur et al., 2004). Miltefosine and AmpB is the current drug of choice with high cure rate, but high toxicity in combination with other adverse side effects and emergence of relapse cases are against these drugs have restrict their use (Pandey et al., 2009; Burza et al., 2014). Previously we have reported that imipramine, a tricyclic antidepressant, is highly effective against antimony-unresponsive clinical LD isolates *in vitro* and *in vivo* in experimental hamster model in full time 28 days oral treatment (Mukherjee et al., 2012). It was also shown that, imipramine acts by generation of leishmanicidal reactive oxygen species (ROS) and nitric oxide (NO) in LD infection and modulates histone deacetylase 11 to increase the IL-12/IL-10 ratio in infected macrophages (Mukherjee et al., 2012) (Mukherjee et al., 2014).It also interferes with sterol synthesis of another leishmania species (*L. amazonensis*) (Andrade-Neto et al., 2016).However, the amount of imipramine needed for ~100% parasite clearance was 140 mg/kg, whereas 50 mg/kg dose clears 90% parasite. Thus, to increase the efficacy of the 10-day short-course imipramine-treatment, we need to deliver increased amount of imipramine within this short span without adverse toxicity, and for this purpose, we used a liposomal formulation of imipramine. In this study, we evaluated the efficacy of liposomal-imipramine against Sb^R^ and Sb^S^LD *in vivo* as oppose to free-Imipramine

Our result showed that formulation of liposomal-imipramine increase antileishmanial activity at low concentration. It is to be noted that this strategy of increasing efficacy and reducing toxicity of anti-leishmanial drug has already showed great promise for polyene antibiotic Amphotericin B and has been commercialized in lipids or liposomal formulations (Barratt and Bretagne, 2007). The superior antileishmanial efficacy of liposomal-imipramine at much lower concentration with 4-day parental treatment as compared to continuousfree-imipramine treatment showed better organ parasite clearance compared to oral alone. Lip-Imi were found to suppress spleen and liver parasite burden almost completely in both mouse and infected hamster model (**Figure 2**).

*Leishmania* infection is associated with decreased IL-12, TNF-α, and IFN-γ production, upregulation of suppressive cytokines like IL-10 and TGF-β, and depressed antigen-specific T-cell response. Macrophage activation occurs through Th1 cytokine response, and IFN-γ plays most important role in it. IFN-γ was found to be significantly elevated in Lip-Imi treated infected mice. According to previous reports, IL-10 surge is higher for infection with Sb^R^LD as compare to sensitive counterparts. Interestingly Liposomal-imipramine treatment was associated with decrease in IL-10 production irrespective of the nature of the input infection (**Figure 3A**, III). It has been recently reported that IL-6 plays an important role in rendering protective immune response against Sb^R^LD mediated infection by lowering IL-10 level (Dey et al., 2020). In the present work, we have also observed that there is a strong suppression of IL-10 level, which might result from increased IL-6 level, as oral imipramine treatment increased IL-6 level in Sb^R^LD mediated infection, and this in all probability will be further enhanced with liposomal imipramine treatment as observed in the increased suppression level of IL-10 in presence of liposomal-imipramine (**Figure 3A**, iv)

Liposomal-imipramine was also found to be highly active to mount anti-SLA specific T-cell expansion compared to oral-imipramine delivery (**Figure 3B**). Thus, treatment with Lip-Imi resulted in clearance of both antimony-susceptible and -resistant strains of LD by switching from release of disease-promoting Th2 (IL-10 and TGF-β) cytokines to disease-resolving Th1 (IL-12 and TNF-α) cytokines coupled with antileishmanial T-cell expansion.

Imipramine is available for chemical use for more than 60 years now. Although it has been associated with toxic side effects including bowels, dizziness, dryness of mouth, headache, etc. with a prolonged treatment at high doses, low dose of imipramine does not exhibit these adverse side effects, and is currently available as prescribed medicine even for children (Saraf and Klein, 1971; People, 2010; Fayez and Gupta, 2020). The liver enzyme levels show decreased liver damage and liposomal imipramine treatment resulted in no significant changes in the hepatic enzyme level as compared to naïve, uninfected animals. Taken together, our observations clearly suggestindicate that the liposomal imipramine formulation has low *in vivo* liver toxicity (**Figure 3C**), although it should be mentioned, which it might take considerable amount of time to bring down hepatic enzyme levels for *Leishmania* infected animals to a comparable level as normal uninfected ones.

The clearance of organ parasite assures better life expectancy of the infected animals. Survival kinetics study showed Sb^R^LD infected hamsters treated with 10 mg/kg of imipramine-liposome remains healthy similar to uninfected animals until the termination of the experiment although all infected hamsters died before the time span (**Figure 4**).

From our work we conclude that liposomal formulation of imipramine is more potent antileishmanial candidate than free-imipramine alone. Treatment with liposomal-imipramine lowered the hepatic/splenic parasite load in both hamster and mice. Furthermore, this formulation promotes the production of host protective cytokines in *in vivo* scenario. *In vivo* testing reported no noticeable toxicity, and levels of the liver enzymes were decreased in mice treated with both imipramine-liposomes compared with mice treated with empty liposomes or free imipramine (**Figure 3C**).

## Data Availability Statement

The original contributions presented in the study are included in the article/supplementary material. Further inquiries can be directed to the corresponding author.

## Ethics Statement

The animal study was reviewed and approved by CSIR-IICB. Written informed consent was obtained from the owners for the participation of their animals in this study.

## Author Contributions

SM performed the experiment, analyzed the data, and prepared the original draft. SP performed the experiment and prepared the original draft. SG performed the experiment and prepared the original draft. SS provided the reagents and materials. SD performed experiment. BM participated in supervision, conceptualization, analyzed the data, preparation of original draft, and editing of the MS. SR participated in supervision, conceptualization, secure funding for the work, analyze the data, preparation of original draft, and editing of the MS. All authors contributed to the article and approved the submitted version.

## Funding

SM received CSIR-NET Fellowship. SP is a recipient of CSIR UGS fellowship. SG is a recipient of IITKGP GATE fellowship. SR is a recipient of JC Bose fellowship.

## Conflict of Interest

The authors declare that the research was conducted in the absence of any commercial or financial relationships that could be construed as a potential conflict of interest.

## References

[B1] Alving C. R. Steck E. A. Chapman W. L. Waits V. B. Hendricks L. D. Swartz G. M. (1978). Therapy of leishmaniasis: superior efficacies of liposome-encapsulated drugs. Proc. Natl. Acad. Sci. 75 (6), 2959–2963. 10.1073/pnas.75.6.2959 208079 PMC392686

[B2] Andrade-Neto V. V. Pereira T. M. do Canto-Cavalheiro M. Torres-Santos E. C. (2016). Imipramine alters the sterol profile in Leishmania amazonensis and increases its sensitivity to miconazole. Parasites Vectors 9 (1), 183. 10.1186/s13071-016-1467-8 27036654 PMC4815111

[B3] Badiee A. Jaafari M. R. Khamesipour A. (2007). Leishmania major: immune response in BALB/c mice immunized with stress-inducible protein 1 encapsulated in liposomes. Exp. Parasitol 115 (2), 127–134. 10.1016/j.exppara.2006.07.002 16979165

[B4] Barratt G. Bretagne S. (2007). Optimizing efficacy of Amphotericin B through nanomodification. Int. J. Nanomed 2 (3), 301–313. PMC2676657 18019830

[B5] Basu R. Bhaumik S. Basu J. M. Naskar K. De T. Roy S. (2005). Kinetoplastid membrane protein-11 DNA vaccination induces complete protection against both pentavalent antimonial-sensitive and -resistant strains of Leishmania donovani that correlates with inducible nitric oxide synthase activity and IL-4 generation: evidence for mixed Th1- and Th2-like responses in visceral leishmaniasis. J. Immunol. 174 (11), 7160–7171. 10.4049/jimmunol.174.11.7160 15905560

[B6] Black C. Watson G. Ward R. J. (1977). The use of Pentostam liposomes in the chemotherapy of experimental leishmaniasis. Trans. R. Soc. Trop. Med. Hyg 71 (6), 550–552. 10.1016/0035-9203(77)90155-9 204085

[B7] Borborema S. E. T. Osso Junior J. A. Andrade Junior H. F. D. Nascimento N. D. (2016). Antimonial drugs entrapped into phosphatidylserine liposomes: physicochemical evaluation and antileishmanial activity. Rev. da sociedade Bras. med Trop. 49 (2), 196–203. 10.1590/0037-8682-0041-2016 27192589

[B8] Burza S. Sinha P. K. Mahajan R. Lima M. A. Mitra G. Verma N. (2014). Risk factors for visceral leishmaniasis relapse in immunocompetent patients following treatment with 20 mg/kg liposomal amphotericin B (Ambisome) in Bihar, India. PloS Negl. Trop. Dis. 8 (1), e2536. 10.1371/journal.pntd.0002536 24392166 PMC3879206

[B9] Clem A. (2010). A current perspective on leishmaniasis. J. Glob Infect. Dis. 2 (2), 124–126. 10.4103/0974-777X.62863 20606967 PMC2889651

[B10] Croft S. L. Coombs G. H. (2003). Leishmaniasis–current chemotherapy and recent advances in the search for novel drugs. Trends Parasitol 19 (11), 502–508. 10.1016/j.pt.2003.09.008 14580961

[B11] Croft S. L. Seifert K. Yardley V. (2006a). Current scenario of drug development for leishmaniasis. Indian J. Med. Res. 123 (3), 399. 16778319

[B12] Croft S. L. Sundar S. Fairlamb A. H. (2006b). Drug resistance in leishmaniasis. Clin. Microbiol Rev. 19 (1), 111–126. 10.1128/CMR.19.1.111-126.2006 16418526 PMC1360270

[B13] Dey S. Mukherjee D. Sultana S. S. Mallick S. Dutta A. Ghosh J. (2020). Combination of Mycobacterium indicus pranii and Heat-Induced Promastigotes Cures Drug-Resistant Leishmania Infection: Critical Role of Interleukin-6-Producing Classical Dendritic Cells. Infect. Immun. 88, e00222–19. 10.1128/IAI.00222-19 32229617 PMC7240079

[B14] Faraut-Gambarelli F. Piarroux R. Deniau M. Giusiano B. Marty P. Michel G. (1997). In vitro and in vivo resistance of Leishmania infantum to meglumine antimoniate: a study of 37 strains collected from patients with visceral leishmaniasis. Antimicrob Agents Chemother. 41 (4), 827–830. 10.1128/AAC.41.4.827 9087498 PMC163803

[B15] Fayez R. Gupta V. (2020). “Imipramine,” in StatPearls (Treasure Island (FL: StatPearls Publishing Copyright © 2020, StatPearls Publishing LLC).

[B16] Fiat W. J. Sanford J. Smith M. W. Spencer V. K. (1990). The assessment and control of the severity of scientific procedures on laboratory animals. Lab. Anim. 24 (2), 97–130. 10.1258/002367790780890185 2366519

[B17] Fox C. B. (2009). Squalene emulsions for parenteral vaccine and drug delivery. Molecules 14 (9), 3286–3312. 10.3390/molecules14093286 19783926 PMC6254918

[B18] Goodman L. S. (1990). Goodman and Gilman’s the pharmacological basis of therapeutics (Pergamon Press New York).

[B19] Gradoni L. Bryceson A. Desjeux P. (1995). Treatment of Mediterranean visceral leishmaniasis. Bull. World Health Organ 73 (2), 191–197. 7743590 PMC2486749

[B20] Guha R. Das S. Ghosh J. Sundar S. Dujardin J. C. Roy S. (2014). Antimony resistant Leishmania donovani but not sensitive ones drives greater frequency of potent T-regulatory cells upon interaction with human PBMCs: role of IL-10 and TGF-β in early immune response. PloS Negl. Trop. Dis. 8 (7), e2995. 10.1371/journal.pntd.0002995 25032977 PMC4102415

[B21] Haldar A. K. Sen P. Roy S. (2011). Use of antimony in the treatment of leishmaniasis: current status and future directions. Mol. Biol. Int. 2011:571242. 10.4061/2011/571242 22091408 PMC3196053

[B22] Hay R. J. (1994). Liposomal amphotericin B, AmBisome. J. Infect. 28 (Suppl 1), 35–43. 10.1016/S0163-4453(94)95956-0 8077689

[B23] Kamimura H. Koga N. Oguri K. Yoshimura H. (1992). Enhanced elimination of theophylline, phenobarbital and strychnine from the bodies of rats and mice by squalane treatment. J. pharmacobio-dynamics 15 (5), 215–221. 10.1248/bpb1978.15.215 1527697

[B24] Klucker M. F. Dalençon F. Probeck P. Haensler J. (2012). AF03, an alternative squalene emulsion-based vaccine adjuvant prepared by a phase inversion temperature method. J. Pharm. Sci. 101 (12), 4490–4500. 10.1002/jps.23311 22941944

[B25] Kohno Y. Egawa Y. Itoh S. Nagaoka S.-I. Takahashi M. Mukai K. (1995). Kinetic study of quenching reaction of singlet oxygen and scavenging reaction of free radical by squalene in n-butanol. Biochim. Biophys. Acta (BBA)-Lipids Lipid Metab. 1256 (1), 52–56. 10.1016/0005-2760(95)00005-W 7742356

[B26] López O. Cócera M. Pons R. Azemar N. López-Iglesias C. Wehrli E. (1999). Use of a dynamic light scattering technique to study the kinetics of liposome solubilization by Triton X-100. Langmuir 15 (13), 4678–4681. 10.1021/la981473a

[B27] Lukes J. Mauricio I. L. Schonian G. Dujardin J. C. Soteriadou K. Dedet J. P. (2007). Evolutionary and geographical history of the Leishmania donovani complex with a revision of current taxonomy. Proc. Natl. Acad. Sci. U.S.A. 104 (22), 9375–9380. 10.1073/pnas.0703678104 17517634 PMC1890502

[B28] Maltezou H. C. (2010). Drug resistance in visceral leishmaniasis. J. BioMed. Biotechnol. 2010:617521. 10.1155/2010/617521 19888437 PMC2771279

[B29] Melby P. C. Chandrasekar B. Zhao W. Coe J. E. (2001). The hamster as a model of human visceral leishmaniasis: progressive disease and impaired generation of nitric oxide in the face of a prominent Th1-like cytokine response. J. Immunol. 166 (3), 1912–1920. 10.4049/jimmunol.166.3.1912 11160239

[B30] Mukherjee S. Mukherjee B. Mukhopadhyay R. Naskar K. Sundar S. Dujardin J. C. (2012). Imipramine is an orally active drug against both antimony sensitive and resistant Leishmania donovani clinical isolates in experimental infection. PloS Negl. Trop. Dis. 6 (12), e1987. 10.1371/journal.pntd.0001987 23301108 PMC3531496

[B31] Mukherjee S. Mukherjee B. Mukhopadhyay R. Naskar K. Sundar S. Dujardin J.-C. (2014). Imipramine exploits histone deacetylase 11 to increase the IL-12/IL-10 ratio in macrophages infected with antimony-resistant Leishmania donovani and clears organ parasites in experimental infection. J. Immunol. 193 (8), 4083–4094. 10.4049/jimmunol.1400710 25217162

[B32] Mukherjee B. Mukherjee K. Nanda P. Mukhopadhyay R. Ravichandran V. Bhattacharya S. N. (2020). Probing the molecular mechanism of aggressive infection by antimony resistant Leishmania donovani. Cytokine 155245. 10.1016/j.cyto.2020.155245 32861564

[B33] Mukhopadhyay R. Mukherjee S. Mukherjee B. Naskar K. Mondal D. Decuypere S. (2011). Characterisation of antimony-resistant Leishmania donovani isolates: biochemical and biophysical studies and interaction with host cells. Int. J. Parasitol 41 (13-14), 1311–1321. 10.1016/j.ijpara.2011.07.013 21920365

[B34] Olliaro P. L. Guerin P. J. Gerstl S. Haaskjold A. A. Rottingen J. A. Sundar S. (2005). Treatment options for visceral leishmaniasis: a systematic review of clinical studies done in Indi -2004. Lancet Infect. Dis. 5 (12), 763–774. 10.1016/S1473-3099(05)70296-6 16310148

[B35] Pandey B. D. Pandey K. Kaneko O. Yanagi T. Hirayama K. (2009). Relapse of visceral leishmaniasis after miltefosine treatment in a Nepalese patient. Am. J. Trop. Med. Hyg 80 (4), 580–582. 10.4269/ajtmh.2009.80.580 19346379

[B36] Pandey R. K. Verma P. Sharma D. Bhatt T. K. Sundar S. Prajapati V. K. (2016). High-throughput virtual screening and quantum mechanics approach to develop imipramine analogues as leads against trypanothione reductase of leishmania. Biomed pharmacother 83, 141–152. 10.1016/j.biopha.2016.06.010 27470561

[B37] PEOPLE N. E. T. M. O. B. I. C. A. Y. (2010). Nocturnal Enuresis: The Management of Bedwetting in Children and Young People (London). 22031959

[B38] Rasch M. R. Bosoy C. A. Yu Y. Korgel B. A. (2012). Chains, sheets, and droplets: assemblies of hydrophobic gold nanocrystals with saturated phosphatidylcholine lipid and squalene. Langmuir 28 (43), 15160–15167. 10.1021/la302734r 23033891 PMC3532054

[B39] Rezazadeh M. Emami J. (2016). A simple and sensitive HPLC method for analysis of imipramine in human plasma with UV detection and liquid-liquid extraction: Application in bioequivalence studies. Res. Pharm. Sci. 11, 168–176. 27168757 PMC4852662

[B40] Romanelli M. M. Costa Silva T. A. Cunha-Junior E. F. Dias Ferreira D. Guerra J. Galisteo A. J. Jr. (2019). Sertraline Delivered in Phosphatidylserine Liposomes is Effective in Experimental Model of Visceral Leishmaniasis. Front. Cell. infect Microbiol. 9, 353. 10.3389/fcimb.2019.00353 31737574 PMC6828611

[B41] Saraf K. Klein D. F. (1971). The safety of a single daily dose schedule for imipramine. Am. J. Psychiatry 128 (4), 483–484. 10.1176/ajp.128.4.483 5098616

[B42] Sundar S. Chatterjee M. (2006). Visceral leishmaniasis - current therapeutic modalities. Indian J. Med. Res. 123 (3), 345–352. 16778315

[B43] Sundar S. Murray H. W. (1996). Cure of antimony-unresponsive Indian visceral leishmaniasis with amphotericin B lipid complex. J. Infect. Dis. 173 (3), 762–765. 10.1093/infdis/173.3.762 8627049

[B44] Sundar S. Makharia A. More D. K. Agrawal G. Voss A. Fischer C. (2000a). Short-course of oral miltefosine for treatment of visceral leishmaniasis. Clin. Infect. Dis. 31 (4), 1110–1113. 10.1086/318122 11049800

[B45] Sundar S. More D. K. Singh M. K. Singh V. P. Sharma S. Makharia A. (2000b). Failure of pentavalent antimony in visceral leishmaniasis in India: report from the center of the Indian epidemic. Clin. Infect. Dis. 31 (4), 1104–1107. 10.1086/318121 11049798

[B46] Sundar S. (2001). Drug resistance in Indian visceral leishmaniasis. Trop. Med. Int. Health 6 (11), 849–854. 10.1046/j.1365-3156.2001.00778.x 11703838

[B47] Thakur C. P. Narayan S. Ranjan A. (2004). Epidemiological, clinical & pharmacological study of antimony-resistant visceral leishmaniasis in Bihar, India. Indian J. Med. Res. 120 (3), 166–172. 15489554

[B48] Valladares J. E. Riera C. González-Ensenyat P. Díez-Cascón A. Ramos G. Solano-Gallego L. (2001). Long term improvement in the treatment of canine leishmaniosis using an antimony liposomal formulation. Vet parasitol 97 (1), 15–21. 10.1016/S0304-4017(01)00389-2 11337123

